# Targeting YAP/TAZ in Combination with PD-L1 Immune Checkpoint Inhibitors in Non-Small Cell Lung Cancer (NSCLC)

**DOI:** 10.3390/cells12060871

**Published:** 2023-03-10

**Authors:** Kostas A. Papavassiliou, Georgios Marinos, Athanasios G. Papavassiliou

**Affiliations:** 1 First University Department of Respiratory Medicine, “Sotiria” Hospital, Medical School, National and Kapodistrian University of Athens, 11527 Athens, Greece; 2 Department of Hygiene, Epidemiology and Medical Statistics, Medical School, National and Kapodistrian University of Athens, 11527 Athens, Greece; 3 Department of Biological Chemistry, Medical School, National and Kapodistrian University of Athens, 11527 Athens, Greece

**Keywords:** Yes-associated Protein (YAP), transcriptional coactivator with PDZ-binding motif (TAZ), non-small cell lung cancer (NSCLC), immune checkpoint inhibitors, programmed cell death protein-1 (PD-1), programmed cell death-ligand 1 (PD-L1)

## Abstract

The survival of non-small cell lung cancer (NSCLC) patients has improved in the last decade as a result of introducing new therapeutics, such as immune checkpoint inhibitors, in the clinic. Still, some NSCLC patients do not benefit from these therapies due to intrinsic resistance or the development of acquired resistance and their malignant disease progresses. Further research on the molecular underpinnings of NSCLC pathobiology is required in order to discover clinically relevant molecular targets that regulate tumor immunity and to develop reasonable therapeutic combinations that will promote the efficacy of immune checkpoint inhibitors. Yes-associated Protein (YAP) and transcriptional coactivator with PDZ-binding motif (TAZ), the final effectors of the Hippo signaling transduction pathway, are emerging as key players in NSCLC development and progression. Herein, we overview studies that have investigated the oncogenic role of YAP/TAZ in NSCLC, focusing on immune evasion, and highlight the therapeutic potential of combining YAP/TAZ inhibitory agents with immune checkpoint inhibitors for the management of NSCLC patients.

## 1. Introduction

Almost one fourth of all cancer-related deaths are attributed to lung cancer and the diagnosis of most patients occurs when the disease has already progressed to an advanced stage. The majority of lung cancer cases falls under the histopathological category of non-small cell lung cancer (NSCLC), which is further divided into adenocarcinoma, squamous cell carcinoma, and large cell carcinoma [1,2]. Recent advances in our understanding of NSCLC pathobiology have led to the development of new therapies, such as targeted therapy and immunotherapy, that have transformed the treatment landscape of patients with advanced NSCLC. Immune checkpoint inhibitors that target programmed cell death protein-1 (PD-1)/programmed cell death-ligand 1 (PD-L1) result in significant improvements in overall survival and progression-free survival [3,4]. Despite the impressive clinical benefit of immune checkpoint inhibitors, some patients will not respond at all and most patients will develop acquired resistance after initially responding to this therapy and ultimately experience disease progression [5]. Therefore, dissecting the mechanisms of immune evasion, resistance to immune checkpoint inhibitors and identifying new targets that can be pharmacologically modulated to promote the efficacy of immunotherapeutic agents in NSCLC are of outmost importance. YAP and TAZ are key transcriptional regulators and the final effectors of the Hippo signaling pathway that have emerged as critical determinants of malignant features, including tumor growth, survival, metastasis, drug resistance, as well as tumor immunity in NSCLC [6,7] (see also Figure no. 1 in Ref. [6]). Developing therapeutic agents that specifically target YAP/TAZ and combine them with immune checkpoint inhibitors in the clinical context of NSCLC may prove to be a very promising therapeutic strategy that will allow us to make further advances in the field of cancer immunotherapy. The present work summarizes the oncogenic role of YAP/TAZ in the pathobiology of NSCLC, primarily focusing on immune evasion, and emphasizes the potential use of simultaneously blocking YAP/TAZ and immune checkpoints as a combinatorial approach for the management of NSCLC patients.

## 2. YAP and TAZ in NSCLC Pathobiology

YAP and TAZ have been characterized as transcriptional co-activators that function as core downstream effectors of the Hippo signaling pathway [8]. Mechanistically, when the Hippo signaling pathway is turned on, the kinases mammalian sterile 20-like kinase 1/2 (MST1/2) phosphorylate and activate large tumor suppressor homolog 1/2 (LATS1/2) kinases, which in turn phosphorylate and inactivate YAP and TAZ by retaining the latter in the cytoplasm and inducing their degradation [8]. On the other hand, when Hippo signaling is downregulated, YAP and TAZ are activated as they become dephosphorylated and translocate to the cell nucleus where they associate with a plethora of transcription factors, particularly TEA domain (TEAD) family members, and trigger the expression of target genes affecting cell growth, metabolism, proliferation, migration, invasion, and apoptosis [8,9].

Research shows that YAP and TAZ participate in the physiological process of lung development, while in the context of normal lung tissue homeostasis, these proteins are not required [10]. In terms of lung cancer, particularly NSCLC, data suggest that numerous malignant features are driven by YAP and TAZ. Increased expression of YAP and TAZ is associated with NSCLC development, progression, as well as a poor prognosis [11,12,13,14,15,16,17,18]. In preclinical studies, the knock-out of the YAP in lung adenocarcinoma mouse models led to a reduction in tumor masses, whereas human NSCLC cells that had YAP or TAZ knocked-down and were injected into nude mice were not able to form tumor masses [19,20]. When comparing metastatic with non-metastatic NSCLC tumors in mouse models, YAP and TAZ nuclear staining was more pronounced in the metastatic tumor tissue [19,20,21]. In studies using human lung cancer tissues, nuclear YAP protein staining was increased when compared to normal lung tissue, while high YAP protein staining in the nucleus was associated with a higher histologic grade and TNM stage (T, tumor; N, nodes; M, metastases), indicating a dismal prognosis in NSCLC patients [13]. These studies suggest that in order for YAP and TAZ to become oncogenic, these proteins must enter the cell nucleus to be able to upregulate the transcription of tumor-promoting genes. This is also supported by studies in human tissues from NSCLC patients where an increased expression of YAP/TAZ-targeted genes was associated with a poor clinical prognosis [16,20].

## 3. YAP and TAZ Regulate PD-L1 Expression

Multiple studies now demonstrate that YAP and TAZ play significant roles in tumor immunity related to various cancers by directly or indirectly regulating the development and functionality of immune cells, including T cells, B cells, macrophages, myeloid-derived suppressor cells (MDSC), and natural killer (NK) cells [7]. We will focus on the modulatory effect of YAP/TAZ on the expression of the immune checkpoint molecule PD-L1 in the setting of lung cancer cells (Table 1). When expressed on the membrane of cancer cells, PD-L1 interacts with its receptor PD-1 to suppress the activation of T-cells, and as a result, cancer cells are able to escape the anti-tumor immune response [22]. In NSCLC cells, YAP was shown to regulate the transcription of PD-L1 by binding to the PD-L1 promoter region [23,24]. Similarly, TAZ was demonstrated to transcriptionally activate PD-L1 expression at its promoter through associating with the TEAD family of transcription factors, and this transcriptional activation of PD-L1 resulted in the apoptosis of T cells.

**Figure 1:**
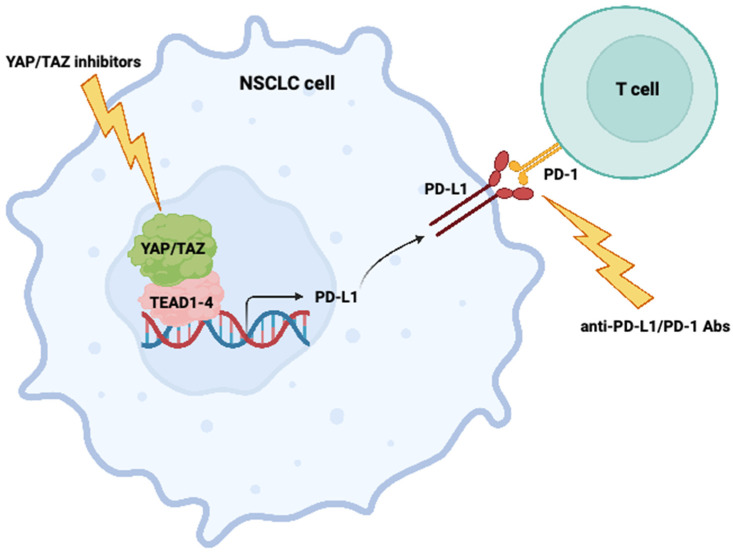
Combining YAP/TAZ inhibitory agents with immune checkpoint inhibitors (e.g., anti-PD-L1/PD-1 antibodies) as a potential therapeutic strategy for NSCLC. This figure was created based on the tools provided by Biorender.com (accessed on 6 March 2023).

Janse van Rensburg et al. presented data suggesting that YAP/TAZ-dependent PD-L1 expression is affected by several known oncogenic signaling pathways, including the epidermal growth factor receptor (EGFR), G-protein-coupled receptor (GPCR), protein kinase C (PKC), 3-phosphoinositide-dependent protein kinase 1 (PDK1), and RAF signaling [25]. In another study, TAZ was shown to transcriptionally upregulate PD-L1 via an upstream mechanism that involves tumor-cell-derived lactate and GPCR81 (also known as hydroxycarboxylic acid receptor 1, HCAR1) signaling [26]. Thus, the transcriptional regulation of PD-L1 by YAP and TAZ may be considered to be confirmative. There are several points that can be inferred from these studies, namely that: (i) TAZ and YAP are able to promote an immunosuppressive environment via the direct upregulation of PD-L1 in NSCLC cells; (ii) TAZ and YAP may be utilized as predictive biomarkers for immune checkpoint inhibitors in NSCLC; and most importantly (iii) Combining TAZ/YAP-targeting agents with immune checkpoint inhibitors may be a promising therapeutic strategy for NSCLC patients (Figure 1).

## 4. YAP/TAZ Pharmacological Targeting

Given the important role of YAP and TAZ in the development and progression of NSCLC, the inhibition of these proteins via pharmacological agents may represent a valid therapeutic opportunity in combination with current therapeutics. What makes the targeting of YAP and TAZ appealing is the interesting fact that they are not required for normal tissue homeostasis. In contrast, YAP and TAZ seem to be necessary for cancer cells to promote their malignant features. Hence, this probably means that inhibiting YAP and TAZ may only affect cancer cells without causing side effects on healthy tissues.

Targeting transcription factors and their co-regulators is a challenging task but it holds great promise as a therapeutic strategy for numerous diseases, including cancer [27,28]. Inhibitors that target YAP and TAZ directly are lacking owing to their particular protein structure. YAP and TAZ are characterized by an intrinsically disordered structure allowing them to be highly flexible [29]. From the perspective of drug development, this means that YAP and TAZ have only few structural regions with a high degree of order that are amenable to pharmacological modulation with small molecule inhibitors. Nevertheless, there are some published data regarding the pharmacological inhibition of YAP and TAZ, which show successful blockade of their oncogenic function in the setting of NSCLC. Guo et al. treated NSCLC cells with norcantharidin (NCTD), a demethylated form of cantharidin, and found that this synthetic compound caused a dose- and time-dependent reduction in both the mRNA and protein levels of YAP, as well as increased YAP phosphorylation. All these effects led to the inhibition of proliferation, invasion, and epithelial-to-mesenchymal transition (EMT), while they promoted senescence and apoptosis in NSCLC cells [12]. The authors of another study used rottlerin, also called mallotoxin, a natural polyphenolic compound isolated from the kamala tree (*Mallotus philippinensis*). When applied to NSCLC cells, rottlerin inhibited cell proliferation, migration, and invasion, as well as induced cell cycle arrest and apoptosis. Results from this study demonstrate that these anti-tumor effects were brought about via a decrease in the protein levels of TAZ [30]. Another strategy to target the activity of YAP and TAZ is to disrupt the physical binding of YAP/TAZ with the TEAD transcription factors. Verteporfin, a benzoporphyrin derivative, is a FDA-approved drug used as a photosensitizer for photodynamic therapy to treat macular degeneration [31]. In terms of its mechanism of action, verteporfin has been shown to block the association between YAP and TEAD, possibly by causing the translocation of YAP to the cytoplasm [32,33]. Several other compounds that disrupt the interaction between YAP and TEAD have been developed; however, none of them have been evaluated in the setting of NSCLC and all of them are currently at the preclinical stage of drug development [34,35,36,37,38,39,40,41].

## 5. Future Perspectives: Targeting the Interaction between YAP/TAZ Signaling and Immune Checkpoints in Cancer

The role of YAP and TAZ in tumor immunity is now emerging and further studies in the future will soon validate their role as key regulators of immune evasion in human cancer. So far, studies suggest that the expression of YAP and TAZ in immune cells, including T and B cells and macrophages, controls their differentiation and function. Furthermore, YAP and TAZ in cancer cells regulate, in an indirect manner, the recruitment and activity of tumor-infiltrating immune cells, as well as the expression of immune checkpoint molecules [7]. Since the transcriptional regulation of PD-L1 by YAP and TAZ has been well established in the setting of NSCLC, as well as in other cancer types [25,42,43], combining YAP- and TAZ-targeting agents with PD-L1/PD-1 inhibitors (e.g., atezolizumab, durvalumab, avelumab, pembrolizumab, and nivolumab) may represent a promising therapeutic strategy in the management of cancer patients. For example, a preclinical study on melanoma combined the YAP/TAZ inhibitor verteporfin with an anti-PD-1 antibody in melanoma-bearing mice and found that this combinatorial treatment suppressed tumor growth to a great extent [44]. Studies exploring this drug combination are still scarce and, thus, if we are to realize its therapeutic potential, additional research is required in this direction. The rationale for such a therapeutic strategy is the following: downregulating the expression of PD-L1 in cancer cells via YAP/TAZ inhibitors reduces the number of available PD-L1 molecules on the cell surface, and so cancer cells have a lower probability of activating the PD-L1/PD-1 axis and evading the anti-tumor immune response. The addition of immune checkpoint inhibitors to this setting lowers even further the chances that cancer cells will escape immune eradication. Moreover, targeting the PD-L1/PD-1 axis at two different points simultaneously does not allow cancer cells to develop acquired resistance that easily.

Interestingly, apart from its role in tumor immunity, PD-L1 was shown to affect cancer cell migration in lung adenocarcinoma cells that are EGFR-tyrosine kinase inhibitor (TKI)-resistant [24]. The authors presented data indicating that YAP transcriptionally regulates PD-L1, and that this signaling axis of YAP/PD-L1 was responsible for the promotion of cancer cell migration independently of PD-1 and T cells. Recent studies also reveal the mechanobiological aspect of PD-L1 in cancer cells, including lung cancer cells [45,46,47]. Mechanotransduction is the process whereby mechanical stimuli emanating from the microenvironment are detected by mechanosensory proteins on the cell surface and, in turn, translated into biochemical signals within the cell that ultimately affect gene expression. Mechanobiology plays a fundamental role in cancer pathobiology, and numerous players that participate in tumor-related mechanotransduction have been identified [48]. Among them, YAP and TAZ have been characterized as key mechanosensors and mechanotransducers in tumor mechanobiology [49,50]. As lung cancer cells depend also on their mechanical properties to promote their malignant features [51,52,53,54], it may be worth probing the potential mechanobiological aspect of the interaction between YAP/TAZ and PD-L1 in NSCLC.

## 6. Conclusions—Outlook

The above considerations indicate that YAP and TAZ play an important role in regulating tumor immunity in NSCLC, particularly through the direct transcriptional control of the immune checkpoint PD-L1, and that the combination of YAP/TAZ-targeting agents with PD-1/PD-L1 inhibitors may be a promising therapeutic strategy in the management of NSCLC patients, which could enhance the efficacy of immune checkpoint inhibitors. By following this therapeutic plan, the same immune checkpoint pathway is being targeted at two different but crucial nodes, hence leaving very little room for cancer cells to develop resistance to this treatment combination. In that vein, this drug combination appears to be effective in other solid tumors (e.g., prostate cancer) [55,56]. Nonetheless, as reported for other cancer types, such as the triple-negative breast cancer with highly suspicious microcalcifications on mammography [57,58], stage-specific molecular signatures/functional biomarkers and the immune system activity status should be taken into account in designing targeted combinatorial immunotherapies for NSCLC.

Future studies should focus on dissecting the molecular architecture of the YAP/TAZ–PD-L1 signaling axis and evaluating the biological and clinical significance of this drug combination in the setting of NSCLC.

## Figures and Tables

**Table 1:** Transcriptional regulation of PD-L1 by YAP and TAZ in NSCLC.

YAP/TAZ	Mechanism	References
YAP	PD-L1 promoter binding	[23,24]
TAZ	PD-L1 promoter binding	[25]
TAZ	PD-L1 promoter binding via lactate/GPCR81/cAMP/PKA signaling	[26]

GPCR81, G-protein-coupled receptor 81; PKA, protein kinase A.

## Data Availability

No new data were created or analyzed in this study. Data sharing is not applicable to this article.
